# Transcriptome Sequencing to Identify Important Genes and lncRNAs Regulating Abdominal Fat Deposition in Ducks

**DOI:** 10.3390/ani12101256

**Published:** 2022-05-13

**Authors:** Chunyan Yang, Zhixiu Wang, Qianqian Song, Bingqiang Dong, Yulin Bi, Hao Bai, Yong Jiang, Guobin Chang, Guohong Chen

**Affiliations:** Key Laboratory of Animal Genetics and Breeding and Molecular Design of Jiangsu Province, Yangzhou University, Yangzhou 225009, China; yangcy202109@163.com (C.Y.); songqianqian8sdau@163.com (Q.S.); bqdong2021@163.com (B.D.); ylbi@yzu.edu.cn (Y.B.); bhowen1027@yzu.edu.cn (H.B.); jiangyong@yzu.edu.cn (Y.J.); gbchang1975@yzu.edu.cn (G.C.)

**Keywords:** duck, abdominal fat deposition, RNA-seq, mRNA, lncRNA

## Abstract

**Simple Summary:**

Abdominal fat deposition affects the quality of duck meat and the feed conversion ratio. Here, we performed transcriptome sequencing of the abdominal adipose tissue of ducks with high and low abdominal fat rate by RNA sequencing, exploring the key regulatory genes and lncRNAs related to abdominal fat deposition. As a result, several candidate genes, lncRNAs, and pathways related to abdominal fat deposition in ducks were retrieved. This study lays the foundations for exploring molecular mechanisms underlying the regulation of abdominal fat deposition in ducks, providing a theoretical reference for breeding high-quality meat-producing ducks.

**Abstract:**

Abdominal fat deposition is an important trait in meat-producing ducks. F2 generations of 304 Cherry Valley and Runzhou Crested White ducks were studied to identify genes and lncRNAs affecting abdominal fat deposition. RNA sequencing was used to study abdominal fat tissue of four ducks each with high or low abdominal fat rates. In all, 336 upregulated and 297 downregulated mRNAs, and 95 upregulated and 119 downregulated lncRNAs were identified. Target gene prediction of differentially expressed lncRNAs identified 602 genes that were further subjected to Gene Ontology and KEGG pathway analysis. The target genes were enriched in pathways associated with fat synthesis and metabolism and participated in biological processes, including Linoleic acid metabolism, lipid storage, and fat cell differentiation, indicating that these lncRNAs play an important role in abdominal fat deposition. *PPAPA*, *FOXO3*, *FASN*, *PNPLA2*, *FKBP5*, *TCF7L2*, *BMP2*, *FGF2*, *LIFR*, *ZBTB16*, *SIRT*, *GYG2*, *NCOR1,* and *NR3C1* were involved in the regulation of abdominal fat deposition. *PNPLA2*, *TCF7L2*, *FGF2*, *LIFR*, *BMP2*, *FKBP5*, *GYG2*, and *ZBTB16* were regulated by the lncRNAs TCONS_00038080, TCONS_0033547, TCONS_00066773, XR_001190174.3, XR_003492471.1, XR_003493494.1, XR_001192142.3, XR_002405656.2, XR_002401822.2, XR_003497063.1, and so on. This study lays foundations for exploring molecular mechanisms underlying the regulation of abdominal fat deposition in ducks and provides a theoretical basis for breeding high-quality meat-producing ducks.

## 1. Introduction

Ducks reared for meat have a short feeding cycle, fast growth rate, high meat production rate, and good meat quality, and occupy an important position among poultry meats. Abdominal fat deposition is a complex quantitative trait and an important economic trait in the duck meat industry. It is closely associated with the animal’s genetic background, developmental stage, and nutritional level. It is regulated by a cascade of genetic factors, including transcription factors, functional genes, long non-coding RNAs (lncRNAs), and adipogenic-related signaling pathways. Fats are composed of a variety of fatty acids that affect meat flavor, pH, tenderness, and juiciness [1]. The abdomen is an important location for the deposition of duck fat. Abdominal fat deposits negatively influence the taste of duck meat and reduce their feed conversion rate, hence affecting the economic benefit to the breeding industry. Therefore, research has focused on effectively controlling abdominal fat deposition in ducks and identifying the underlying genetic mechanisms.

LncRNAs are a class of non-coding RNAs with lengths greater than 200 bp [2] and almost no protein-coding capability [3]. Most lncRNAs have significant spatio-temporal expression specificity [4,5], low sequence conservation across species [6,7], and can directly or indirectly regulate adipogenesis [8]. They play an important regulatory function in various biological processes, including adipocyte differentiation and lipid metabolism [9,10,11]. With breakthroughs and innovations in high-throughput sequencing technologies, more lncRNAs have been shown to regulate fat metabolism at the epigenetic, transcriptional, and post-transcriptional levels [12,13]. Transcriptome sequencing (RNA-seq) is a useful tool for transcriptomic analysis that can be used to study molecular mechanisms from the perspective of multi-gene network regulation, leading to the identification of structural genomic changes, gene fusion events, and novel genes and transcripts [14]. RNA-seq has high sensitivity when it comes to identifying differentially expressed genes and quantitatively analyzing transcriptomes [15] and has been widely used in the identification and functional analysis of lncRNAs and mRNAs in adipose tissue of pigs [16,17], chickens [18], sheep [5,19], and cattle [20].

There has been a recent increase in the use of RNA-seq analysis to identify key lncRNAs that regulate animal adipose tissue. However, multiple lncRNAs associated with fat metabolism in ducks have not yet been identified, and the relationship between some lncRNAs and their potential target genes is not clear. Here, the F2 generation of Cherry Valley Duck × Runzhou Crested White Duck cross was studied. RNA-seq data was used to analyze the abdominal fat tissue of four ducks with high abdominal fat rate (HF) and four ducks with low abdominal fat rate (LF) to identify important genes and lncRNAs that regulate abdominal fat deposition in ducks. This study provides a basis for the identification of the molecular mechanism underlying the regulation of duck abdominal fat deposition and provides an experimental basis for the protection, rearing, and utilization of ducks.

## 2. Materials and Methods

### 2.1. Animals and Study Samples

In this study, 304 F2 generation of Cherry Valley Duck × Runzhou Crested White Duck, composed of 162 male ducks and 142 female ducks, were purchased from Shuyang Zhongke Seed Poultry Co., Ltd. (Suqian, China). The ducks were reared in the same batch and under the same conditions for 42 days before being slaughtered according to NY/T823-2020 “Poultry Production Performance Terminology and Measurement Statistical Methods”. The body weight, whole evisceration weight, and abdominal fat weight were measured, and the abdominal fat rate calculated.

### 2.2. Total RNA Extraction and Sequencing

Total RNA was extracted from abdominal adipose tissue using the Trizol reagent kit (Invitrogen, Carlsbad, CA, USA) following the manufacturer’s instructions. RNA concentration and quality were measured at OD 260/280 using the Nanodrop ND-2000 ultra-micro spectrophotometer (Thermo Fisher Scientific, Waltham, MA, USA). The OD260/OD280 (Ratio, R) of the RNA was between 1.8 and 2.0 and the concentration was over 500 ng/µL. The integrity of the RNA was measured by analyzing 2 µL of the total RNA on a 1% agarose gel. The PrimeScript RT reagent Kit with gDNA Eraser was used to reverse transcribe the RNA to generate cDNA following the manufacturer’s instructions. A cDNA library was then constructed and sequenced on the Illumina HiSeqTM 4000.

### 2.3. Data Processing and Analysis

Fastp (0.18.0) [21] was used to control and filter the raw read data. Clean reads were obtained after removing reads containing adapters, reads with an N ratio exceeding 10%, reads with all A bases, and low-quality reads. The reads were filtered further to obtain high-quality clean reads. Bowtie 2 (2.2.8) [22] was used to align the high-quality clean reads against species-specific ribosomal sequences and sequences that aligned to ribosomal RNA were removed. The reads that were filtered out of ribosomal RNA were aligned to the duck reference genome (CAU-Wild 1.0) using the alignment software Tophat2 (2.1.1). Cufflink (2.1.1) software [23] was used to assemble transcripts based on the Tophat2 alignment results. Multiple sequence assemblies were merged using cuffmerge and filtered to generate unique annotation files for transcripts that may have been artificially introduced with assembly errors for subsequent differential analysis. Transcripts were further screened to obtain lncRNA information. Coding Potential Calculator (CPC, v0.9-r2) [24] and Coding-Non-Coding Index (CNCI, v2.0) [25] software were used to predict the coding abilities of new transcripts. The new transcripts were then aligned to the SwissProt protein database (https://ngdc.cncb.ac.cn, accessed on 18 February 2022) and the intersection of the results from the three software was taken as the identified lncRNAs.

### 2.4. Analysis of Differential Expression

Data-normalized quantification of FPKM (expected number of fragments per kilobase of transcript sequence per millions base pairs sequenced) for each individual duck was performed using StringTie (v1.3.1) software [26]. Differential expression analysis was performed using DESeq2 software [27], with fold change (FC, fold of difference) and *p* values being used to measure statistical significance. Among the high and low abdominal fat rate groups, mRNAs whose expression levels were associated with FC ≥ 2 and *p* ≤ 0.01 were considered differentially expressed mRNAs, while lncRNAs whose expression levels were associated with FC ≥ 2 and *p* ≤ 0.05 were considered differentially expressed.

### 2.5. Analysis of Gene Ontology and KEGG Pathway Enrichment

Gene Ontology (GO) is widely used in bioinformatics to analyze gene function from three aspects: Cellular Component (CC), Molecular Function (MF), and Biological Process (BP). Kyoto Encyclopedia of Genes and Genomes (KEGG) is a database for analyzing gene function and genomic information, allowing the study of genes and gene expression information as a whole network. In this study, GO annotation and KEGG functional enrichment analysis of differentially expressed mRNAs and lncRNAs were performed.

### 2.6. Combined mRNA and lncRNA Analysis

LncRNAs are involved in the regulation of many post-transcriptional processes, regulating target genes through antisense, cis, and trans effects. Similar to small RNAs such as miRNA and snoRNA, this regulation is often associated with complementary base pairing. Target genes were predicted using correlation or co-expression analysis of lncRNA and protein-coding genes. To show the interaction between lncRNA and mRNA, correlation of differentially expressed mRNAs and lncRNAs was performed to predict antisense, cis, and trans target genes.

### 2.7. Validation of DEGs Using Quantitative Real Time PCR (qRT-PCR)

qRT-PCR was used to verify the levels of expressed genes. Eight differentially expressed genes (four mRNAs and four lncRNAs) were randomly selected for validation (Table 1), with glyceraldehyde-3-phosphate dehydrogenase (*GAPDH*) as the internal reference gene. PowerUpTM SYBRTM Green Master Mix (A25742, Thermo Fisher, Beijing, China) and the LightCycler 96 Real-Time PCR Detection System (Roche, Basel, Switzerland) were used for qRT-PCR. A 20 µL reaction volume consisting of 10 µL PowerUp™ SYBR™ Green Master Mix (2X), 0.8 µL Forward Primer, 0.8 µL Reverse Primer, 2 µL cNDA template, and 6.4 µL ddH_2_O was used. The following qRT-PCR conditions were used: denaturation at 95 °C for 10 min, followed by 40 cycles of denaturation at 95 °C for 3 s, annealing at 50–60 °C for 30 s, and elongation at 72 °C for 20 s. Each sample was analyzed in triplicate. 

### 2.8. Statistical Analysis

Excel 2019 software was used for data analysis. Results are expressed as mean ± standard deviation (Mean ± SD). Gene expression was calculated using the relative quantification (2^−ΔΔCT^) method. The t-test was used for pairwise analysis in SPSS 22.0 (SPSS, Inc., Chicago, IL, USA). *p* < 0.05 was used to represent statistically significant differences. GraphPad Prism 9.0 and Lianchuan biological cloud platform were used for generating maps.

## 3. Results

### 3.1. Selection of Individual Ducks

Analysis of 304 F2 generation of Cherry Valley × Runzhou Crested White duck crosses showed that the distribution of abdominal fat was associated with gender, and there were differences between ducks with high and low abdominal fat rates. About two-thirds of the ducks had abdominal fat rates of 0.75–1.5%. The 42 d F2 generation ducks with 0–0.75% and 1.5–2.25% abdominal fat rates were classified as having low and high abdominal fat rates, respectively. Differences between these two groups were statistically significant (*p* < 0.05). Four F2 generation male ducks in the high abdominal fat rate range and four in the low abdominal fat rate ranges were randomly selected for slaughter and extraction of abdominal fat tissue (Table 2).

### 3.2. Sequence Data Quality Statistics

The quality control of the sequence data from each sample is shown in Table 3. More than 80 M clean reads were obtained, with the proportion of high-quality clean reads for each sample being greater than 99.2%. A total of 734 M clean reads were obtained from eight samples. The read length was 150 + 150. The proportion of reads with Q20 was greater than 98%, while the proportion of reads with Q30 was greater than 94%. The reads aligned to the duck reference genome are shown in Table 4. The alignment rate of each sample was more than 80%. The sequence data met the requirements for bioinformatics analysis.

### 3.3. Screening and Identification of lncRNAs

**Figure 1:**
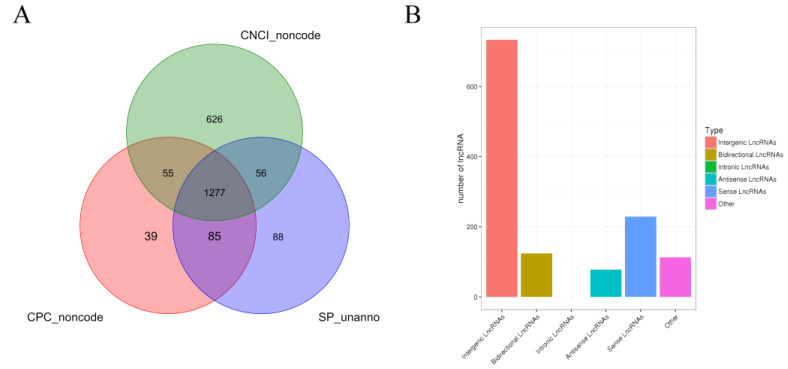
Identification and analysis of lncRNA. Note: (**A**) Venn diagram of annotation results of CPC, CNCI, and Swissprot. (**B**) Statistical chart of new lncRNA transcript types.

A statistical summary of known and new mRNAs and lncRNAs in each sample is shown in Table 5. A total of 11,943 new transcripts were obtained by comparing the results and the length and position of the transcripts. CPC, CNCI, and SwissProt software were used to predict new lncRNAs of these new transcripts, and 1277 new lncRNAs were identified (Figure 1A). Based on the position of the new lncRNAs on the genome relative to the protein-coding genes, the identified lncRNAs were classified into five categories: 733 (57.4%) intergenic lncRNAs, 124 (9.7%) bidirectional lncRNA, 78 (6.1%) antisense lncRNA, 229 (17.9%) sense overlapping lncRNAs, and 113 (8.8%) other types of lncRNAs (Figure 1B).

### 3.4. Analysis of Differentially Expressed Genes

**Figure 2:**
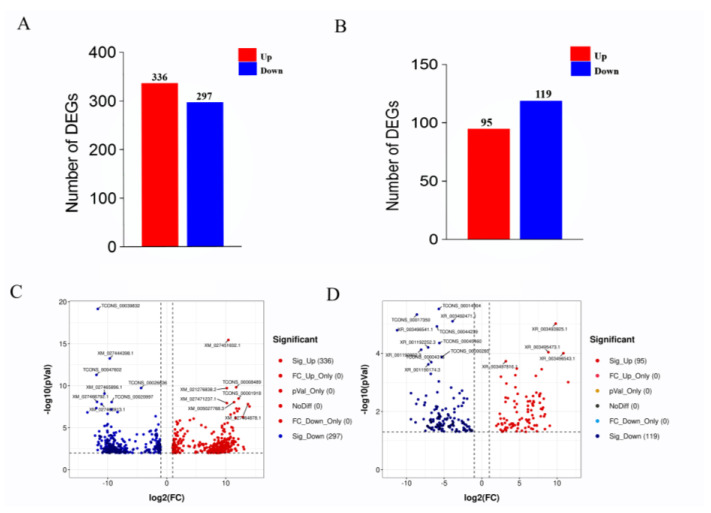
The expression of differential mRNA (DEGs) and differential lncRNA (DELs) in the high and low abdominal fat rate groups. Note: (**A**) Statistics on the number of DEGs in the high and low abdominal fat rate groups; (**B**) statistics on the number of DELs in the high and low abdominal fat rate groups; (**C**) volcano map of DEGs, the *y* axis is the value of −log10 (*p* Value), the *x* axis is the value of log2 (FC), and the two threshold lines respectively represent *p* = 0.01 and FC = 2; (**D**) volcano map of DELs, the *y* axis is the value of −log10 (*p* Value), and the *x* axis is the value of log2 (FC). The two threshold lines respectively represent FDR = 0.05 and FC = 2.

High-throughput sequencing and related bioinformatic analysis were used to identify 39,904 mRNA transcripts, including 29,134 known and 8688 new transcripts. LncRNA and mRNA transcript expression profiles were analyzed for differential expression using Deseq2 (v1.6.3). mRNAs that were differentially expressed between the HF and LF groups were identified based on *p* ≤ 0.01 and |log2FC| ≥ 1 and lncRNAs that were differentially expressed between the HF and LF groups were identified based on *p* ≤ 0.05 and |log2FC| ≥ 1. A total of 633 differentially expressed mRNAs were identified, of which 336 were significantly upregulated and 297 genes were significantly downregulated (Figure 2A,C). We also identified 214 differentially expressed lncRNAs, of which 95 were significantly upregulated and 119 were significantly downregulated (Figure 2B,D).

### 3.5. Functional Annotation of Differentially Expressed Genes (DEGs)

**Figure 3:**
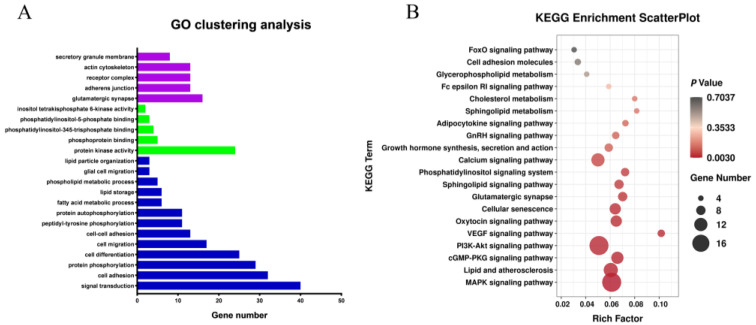
Analysis of Gene Ontology and KEGG Pathway Enrichment of differential mRNAs. Note: (**A**) Gene Ontology of differential mRNAs. Blue column represents BP, red column represents CC and green column represents MF; (**B**) KEGG Pathway Enrichment of differential mRNAs.

Functional annotation was conducted to obtain a deeper understanding of the DEGs (Figure 3A,B). GO analysis showed that most of the differentially expressed genes were enriched in lipid particle organization, cellular fat metabolism, fatty acid metabolic process, lipid storage, and other GO terms associated with fat metabolism. The genes enriched in these GO terms included *AASDH*, *FASN*, *EHHADH*, *NAAA*, *PPARA*, *ACSBG2*, *DGAT2*, *ACVR1C*, *CIDEA*, *PLIN3*, *APOA1*, *PNPLA2,* and so on. KEGG pathway enrichment analysis showed that most of the differentially expressed genes were enriched in Lipid and atherosclerosis, Cell adhesion molecules, Sphingolipid metabolism and other fat metabolism-related pathways, as well as MAPK, Calcium, GnRH, and other growth-related pathways, indicating that the process of fat metabolism is accompanied by growth and other life activity. Genes involved in the KEGG signaling pathways include *LYN*, *APAF1*, *SRC*, *APOA1*, *NFATC1*, *VLDLR*, *PTK2*, *NFKB1*, *ERN1,* and so on. NCBI gene function annotation was also used to screen out *FASN*, *FGF2*, *HIPK3*, *LIFR*, *JCD*, *GYGR*, *FKBP5*, *TSPAN15,* etc., candidate genes associated with abdominal fat deposition.

### 3.6. Analysis of Association and Prediction of Target Gene Function

Target genes of differentially expressed lncRNAs were predicted through antisense, cis, and trans effects to obtain lncRNA–mRNA target gene pairs. Further correlation analysis showed that 218 differentially expressed lncRNAs were associated with 602 target genes, of which one differentially expressed lncRNA and one differentially expressed target gene had co-expression regulation (antisense). Furthermore, 9 differentially expressed lncRNAs and 7 differentially expressed target genes were cis-regulated; 208 differentially expressed lncRNAs and 594 differentially expressed target genes were trans-regulated. These results show that most of the differentially expressed lncRNAs regulate target genes via trans-regulation.

**Figure 4:**
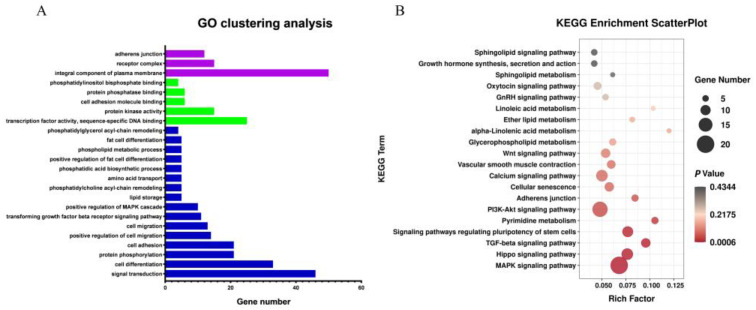
Analysis of Gene Ontology and KEGG Pathway Enrichment of target genes of differential lncRNAs. Note: (**A**) Gene Ontology of target genes of differential lncRNAs. Blue column represents BP, red column represents CC, and green column represents MF; (**B**) KEGG Pathway Enrichment of target genes of differential lncRNAs.

GO and KEGG pathway analyses were conducted on differentially expressed target genes and their corresponding differentially expressed lncRNAs (Figure 4A,B). GO analysis results showed that most of the differentially expressed target genes were associated with several GO terms, including fat cell differentiation, protein kinase activity, lipid storage, and phosphatidylglycerol acyl-chain remodeling. The target genes enriched on these GO terms included *TCF7L2*, *NR4A3*, *WNT5B*, *FAM120B*, *ADGRF5,* and so on. KEGG pathway enrichment analysis showed that most of the differentially expressed target genes were enriched in Glycerophospholipid metabolism, Linoleic acid metabolism, ether lipid metabolism, GnRH signaling pathway, MAPK signaling pathway, Calcium signaling pathway, Wnt signaling pathway and other fat metabolism-related pathways. The target genes enriched in the KEGG pathways included *TLE3*, *TCF7L2*, *PLCB4*, *SFRP2*, *PPP3CC*, *WNT5B*, *MYC*, *DVL3*, *NFATC1,* and so on. Additional NCBI gene function annotation screening identified *BMP2*, *GYG*, *TCF7L2*, *PDZD2*, *SOD3*, *FOXO3*, *TSPAN4*, *LIFR,* and so on.

### 3.7. Protein–Protein Interactions of Target Genes

**Figure 5:**
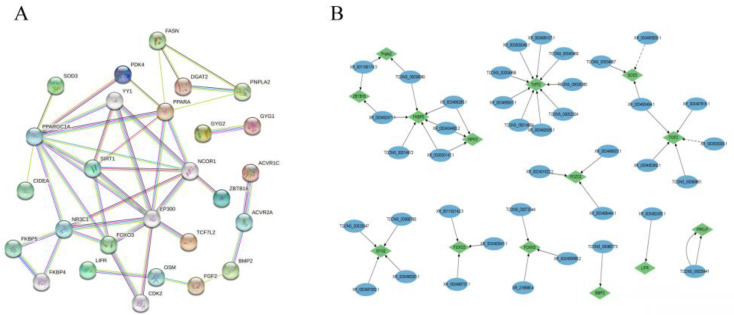
The interaction between DE lncRNA and its target genes as well as DEGs. Note: (**A**) Protein–protein interactions of genes and target genes of differential lncRNAs. Note: (**B**) Interactions between lncRNAs and target genes.

Protein–protein interactions of candidate genes and target genes involved in abdominal fat deposition were performed using the String online software (https://cn.string-db.org, accessed on 18 February 2022). *PPAPA*, *FOXO3*, *GYG2*, *FASN*, *PNPLA2*, *FKBP5*, *TCF7L2*, *BMP2*, *FGF2*, *LIFR*, *ZBTB16*, *SIRT*, *NCOR1,* and *NR3C1* were highly correlated with other genes (Figure 5A), indicating that these genes interact with each other to regulate abdominal fat deposition, with *PNPLA2*, *TCF7L2*, *FGF2*, *LIFR*, *BMP2*, *FKBP5*, *GYG2,* and *ZBTB16* being regulated by lncRNAs TCONS_00038080, TCONS_0033547, TCONS_00066773, XR_001190174.3, XR_003492471.1, XR_003493494.1, XR_001192142.3, XR_002405656.2, XR_002401822.2, and XR_003497063.1 (Figure 5B).

### 3.8. Validation of DEGs

**Figure 6:**
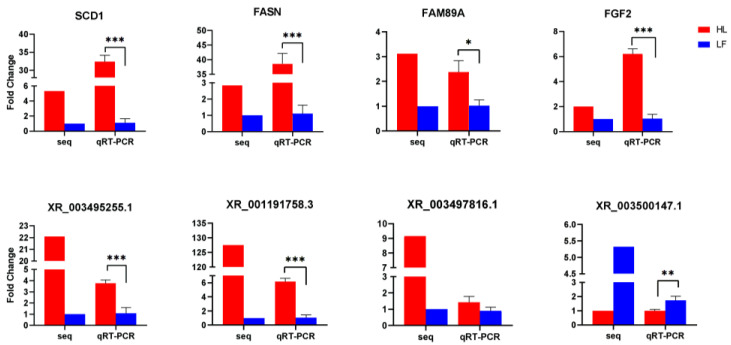
qRT-PCR verification of differentially expressed genes/lncRNA results. Red represents HL and blue represents LF (0.01 < * *p* < 0.05, ** *p* < 0.01, *** *p* < 0.001).

Eight randomly selected differentially expressed genes and lncRNAs were subjected to qRT-PCR to verify the RNA sequencing results from the high and low abdominal fat rate groups. When we performed ANOVA analysis on the qRT-PCR results, we undertook the homogeneity of variance test. The qRT-PCR results of all genes and lncRNAs were in line with the homogeneity of variance. The F and *p* values of each gene and lncRNA are listed in the Supplementary Table S8. The trends of the expression of the four differentially expressed genes and the four differentially expressed lncRNAs in the high and low abdominal fat rate groups were consistent with the RNA sequencing results (Figure 6), indicating that the DEGs identified using the RNA-seq approach were reliable.

## 4. Discussion

Abdominal fat deposition is an important economic trait in meat-producing ducks, and it is therefore important to explore the genes and lncRNAs that regulate abdominal fat deposition in ducks. We used RNA sequencing to generate gene expression profiles of abdominal adipose tissue. GO and KEGG enrichment analysis showed that differentially expressed genes were mainly involved in GnRH, PPAR, and VEGF signaling pathways as well as in biological processes such as fatty acid metabolism and adipocyte differentiation. *PPAPA*, *FOXO3*, *FASN*, *PNPLA2*, *FKBP5*, *TCF7L2*, *BMP2*, *FGF2*, *LIFR*, *ZBTB16*, *SIRT*, *GYG2*, *NCOR1,* and *NR3C1* were involved in the regulation of abdominal fat deposition. *PNPLA2*, *TCF7L2*, *FGF2*, *LIFR*, *BMP2*, *FKBP5*, *GYG2,* and *ZBTB16* were regulated by differentially expressed lncRNAs.

*PPARA* is a member of the PPAR family. In the field of molecular biology [28], PPARA is a nuclear receptor protein, mainly involved in the regulation of transcription factor expression, and plays an important role in cell differentiation, development, and metabolism. PPARα regulates lipid transport and metabolism through the peroxisomal fatty acid β-oxidation pathway [29]. A critical role for PPARA in fat accumulation and binding was identified in a mouse study by Mohamed et al. [30]. We therefore hypothesized that *PPAPA* is involved in regulating abdominal fat deposition.

*FOXO1* and *FOXO3* belong to the *FOXO* gene family. *FOXO1* can inhibit adipogenesis by reducing the transcriptional activity of PPARγ [31,32]. It can also affect adipogenesis by regulating the expression of *MAF1* and *SREBPs* that are involved in adipogenesis and lipophagy [33]. *FOXO1* protein is an inhibitor of uncoupling protein 1 (*UCP1*) gene transcription [34]. Decreased *FOXO1* protein expression in adipose tissue leads to thermogenesis of adipose tissue, one of the causes of increased energy expenditure and weight loss [35]. These studies showed that *FOXO1* is associated with fat metabolism [36,37]. Although *FOXO1* did not affect abdominal fat deposition in ducks in this study, *FOXO3* was differentially expressed in the HF group. Therefore, the role of *FOXO3* in fat metabolism needs to be explored further.

*FASN*, a multi-enzyme complex comprising seven enzymes with different functions and an acyl carrier protein (ACP), is critical in fatty acid synthesis [38,39,40] and fatty acid biosynthesis [41,42]. In this study, we found that *FASN* expression is associated with abdominal fat deposition. Berndt et al. found that *FASN* expression significantly correlated with obesity and hinted that specific inhibition of *FASN* could represent a new way to prevent and treat obesity and its complications [43].

*PNPLA2*, a lipase encoding fatty triglycerides responsible for catalyzing the breakdown of triglycerides (TAGs) [44], was first identified as the major TAG lipase in adipose and cardiac tissues in 2004 [45]. In the liver, genetic ablation of *PNPLA2* promotes steatosis [46,47,48]. Turpin et al. found that *PNPLA2* overexpression in the liver attenuated steatosis [49].

*FKBP5* was highly expressed in human skeletal muscle and adipose tissue [50]. Sidibeh et al. showed that *FKBP5* gene expression is inversely correlated with the expression of lipogenesis and lipolysis and related lipogenic genes [51]. Therefore, the role of *FKBP5* in abdominal fat deposition needs to be explored further.

Previous studies have shown that *TCF7L2* is involved in regulating adipocyte differentiation, triglyceride hydrolysis, and lipogenesis. Dominant inactivation of *TCF7L2* promotes adipogenesis [52]. Kaminska et al. have shown that short *TCF7L2* mRNA variants are associated with body weight and hyperglycemia and insulin in subcutaneous adipose tissue [53]. *TCF7L2* actively represses Wnt-responsive genes during adipocyte differentiation through direct interaction with TLE3 [54]. Chen et al. showed that *TCF7L2* knockout impairs adipocyte differentiation [55], affecting fat metabolism. Geoghegan et al. showed that *TCF7L2* is highly expressed in white fat and directly regulates cellular metabolism-related genes and demonstrated that adipocyte-specific conditional deletion of *TCF7L2* results in adipocyte hypertrophy [56].

Bone morphogenetic protein 2 (*BMP2*) is a classic morphogen, a molecule that acts at a distance to promote adipogenic and osteogenic differentiation [57] and is involved in the regulation of fat [58]. Lu et al. found that *BMP2* overexpression in sheep preadipocytes can promote adipogenic differentiation [59]. *BMP2* can also promote the formation of adipocytes in the white pre-adipose cell lines 3T3-L1 [60], A33 [58], and 3T3-F442A [61], but when combined with retinoic acid, *BMP2* inhibited the formation of adipocytes in 3T3-F442A cells [62]. In this study, *BMP2* was differentially expressed in F2 generation ducks with high and low abdominal fat rates, and we hypothesized that it was involved in the regulation of abdominal fat deposition.

Fibroblast growth factor 2 (*FGF2*) is one of the first recognized members of the FGF family [63]. We found that *FGF2* is associated with abdominal fat deposition, although other studies have shown that *FGF2* is involved in the regulation of white adipogenic differentiation [64,65,66,67]. Kawaguchi et al. demonstrated the induction of de novo lipogenesis in recombinant basement membrane supplemented with *FGF2* [64]. *FGF2* significantly enhanced adipogenic differentiation and PPARγ expression of human adipose stem cells (hASC) [65] while Xiao et al. found that mice lacking *FGF2* enhanced the adipogenesis ability of bone marrow stem cells [67]. Additionally, disrupting *FGF2* increased the thermogenic capacity of brown and beige fat and *FGF2* loss protected mice from high-fat-induced obesity and hepatic steatosis [68].

In addition to the above genes, which have been confirmed to be involved in the synthesis and metabolism of abdominal fat, this study also showed that *LIFR*, *GYG2*, *ZBTB16*, *SIRT*, *NCOR1,* and *NR3C1* may be involved in the regulation of abdominal fat deposition in ducks. *PNPLA2*, *TCF7L2*, *FGF2*, *LIFR*, *BMP2*, *FKBP5*, *GYG2,* and *ZBTB16* were regulated by the differentially expressed lncRNAs TCONS_00038080, TCONS_0033547, TCONS_00066773, XR_001190174.3, XR_003492471.1, XR_003493494.1, XR_001192142.3, XR_002405656.2, XR_002401822.2, XR_003497063.1, and so on. We hypothesized that these lncRNAs regulate the production of abdominal fat by regulating the specified genes.

## 5. Conclusions

RNA-seq was used to study the abdominal fat tissue of four ducks in high and low abdominal fat rate groups. Eleven key candidate genes *PPAPA*, *FOXO3*, *FASN*, *PNPLA2*, *FKBP5*, *TCF7L2*, *BMP2*, *FGF2*, *LIFR*, *ZBTB16*, *SIRT*, *GYG2*, *NCOR1* and *NR3C1,* and key lncRNAs TCONS_00038080, TCONS_0033547, TCONS_00066773, XR_001190174.3, XR_003492471.1, XR_003493494.1, XR_001192142.3, XR_002405656.2, XR_002401822.2, XR_003497063.1, etc., were identified using differential expression and bioinformatic analyses. The results of this study lay a foundation for exploring the molecular mechanisms underlying the regulation of abdominal fat deposition in ducks and provide a theoretical basis for breeding meat-producing ducks and high-quality livestock and poultry products with greater economic benefits.

## Figures and Tables

**Table 1:** Primer information.

Genes/lncRNA	Primer Sequences (5′ → 3′)	Product Length/bp	Annealing Temperature/°C
SCD1	F:TATTGCAAACTCCGTGGCCT R:AGGGCTTGTAGTATTTCCGCT	245	59
FASN	F:CCAGCAAATCAGCTCATGCC R:TCACGTCTCGGACACCAATG	167	60
FAM89A	F:TCCGCAAGGAGATGGTTGG R:TACGTGCAGTCTGCGTTAGA	127	59
FGF2	F:CTGTACTGCAAGAACGGCGG R:TCTTCTGTTGCGCATTTCAGT	206	60
XR_003497816.1	F:TTCCGAAAACTGAGCCCGAA R:TTCCGAAAACTGAGCCCGAA	172	59
XR_001191758.3	F:GCCCAGAACTGAAACCAAGC R:TGGCCTGTTTCACGACAGAT	147	59
XR_003500147.1	F:TTCCTCTTTTCACTGGCGCT R:GTGACCATCCATCAGGTGGG	214	60
XR_003495255.1	F:TGAGCTGGCCTTTCCAGATG R:AACCTTGCCACGTAAACCCA	239	60
GAPDH	F:GGTTGTCTCCTGCGACTTCA R:TCCTTGGATGCCATGTGGAC	116	60

**Table 2:** Distribution of abdominal fat percentage in F2 generation ducks.

Abdominal Fat Rate (%)	Quantity	Number of Drakes	Number of Female Ducks	Male/Female Duck
Abdominal fat rate ≥ 1.75	19	2	17	0.1176
1.50 ≤ Abdominal fat rate < 1.75	49	17	32	0.5313
0.75 ≤ Abdominal fat rate < 1.5	221	124	97	1.4639
Abdominal fat rate < 0.75	34	21	13	1.6154
Total	304	162	142	1.1409

**Table 3:** Filtered information statistics table of reads.

Sample	Clean Reads Num	HQ Clean Reads Num (%)	Read Length	Q20 (%)	Q30 (%)
HF-1	87,815,840	99.23%	150 + 150	98.43%	94.77%
HF-2	67,642,082	99.1%	150 + 150	98.53%	95.07%
HF-3	80,074,662	99.24%	150 + 150	98.34%	94.49%
HF-4	87,797,784	99.3%	150 + 150	98.54%	95.08%
LF-1	64,877,042	99.68%	150 + 150	98.20%	94.20%
LF-2	98,580,674	99.16%	150 + 150	98.13%	93.97%
LF-3	85,446,600	99.3%	150 + 150	98.57%	95.15%
LF-4	90,275,540	99.27%	150 + 150	98.40%	94.65%

**Table 4:** Comparison of gene statistics.

Sample	Total Reads	Unmapped Reads	Unique Mapped Reads	Multiple Mapped Reads	Mapping Ratio
HF-1	78,360,250	13,256,692	64,460,250	643,308	83.08%
HF-2	50,275,220	9,793,210	40,152,834	329,176	80.52%
HF-3	67,770,884	11,466,904	55,696,058	607,922	83.08%
HF-4	66,524,018	11,225,437	54,740,395	558,186	83.13%
LF-1	45,459,676	9,181,060	35,978,304	300,312	79.80%
LF-2	94,305,132	15,695,628	77,878,940	730,564	83.36%
LF-3	72,114,628	11,324,254	60,240,554	549,820	84.30%
LF-4	82,004,742	12,254,346	69,035,082	715,314	85.06%

**Table 5:** Summary of transcript statistics.

Sample Name	Known mRNA Num	New mRNA Num	All mRNA Num	Known lncRNA Num	New lncRNA Num	All lncRNA Num
HF-1	21,297	6281	27,578	2497	794	3291
HF-2	20,102	6032	26,134	2370	736	3106
HF-3	21,156	6279	27,435	2318	748	3066
HF-4	20,999	6221	27,220	2321	764	3085
LF-1	19,880	5923	25,803	2205	718	2923
LF-2	21,535	6435	27,970	2577	802	3379
LF-3	21,278	6284	27,562	2504	811	3315
LF-4	21,069	6317	27,386	2491	774	3265

## Data Availability

The data presented in this study are available on request from the corresponding author.

## Supplementary Materials

The following are available online at https://www.mdpi.com/article/10.3390/ani12101256/s1, Supplementary Figure S1: lncRNA transcript coverage statistics, Figure S2: mRNA transcript coverage statistics, Table S3: Differential mRNA statistics, Table S2: Differential lncRNA statistics, Table S3: Differential lncRNA target gene statistics, Table S4: Differential mRNA GO analysis, Table S5: KEGG analysis of differential mRNA GO analysis, Table S6: GO analysis of target genes, Table S7: KEGG analysis of target genes, Table S8: Analysis results of qRT-PCR.
