# Supplementary material for: Transcriptome Sequencing to Identify Important Genes and lncRNAs Regulating Abdominal Fat Deposition in Ducks
Source: Animals (Basel). 2022 May 13;12(10):1256. doi: 10.3390/ani12101256 (PMC9138122; doi:10.3390/ani12101256)
Supplement: Supplementary file 1 [file animals-12-01256-s001.zip › Supplementary Figures.pdf]

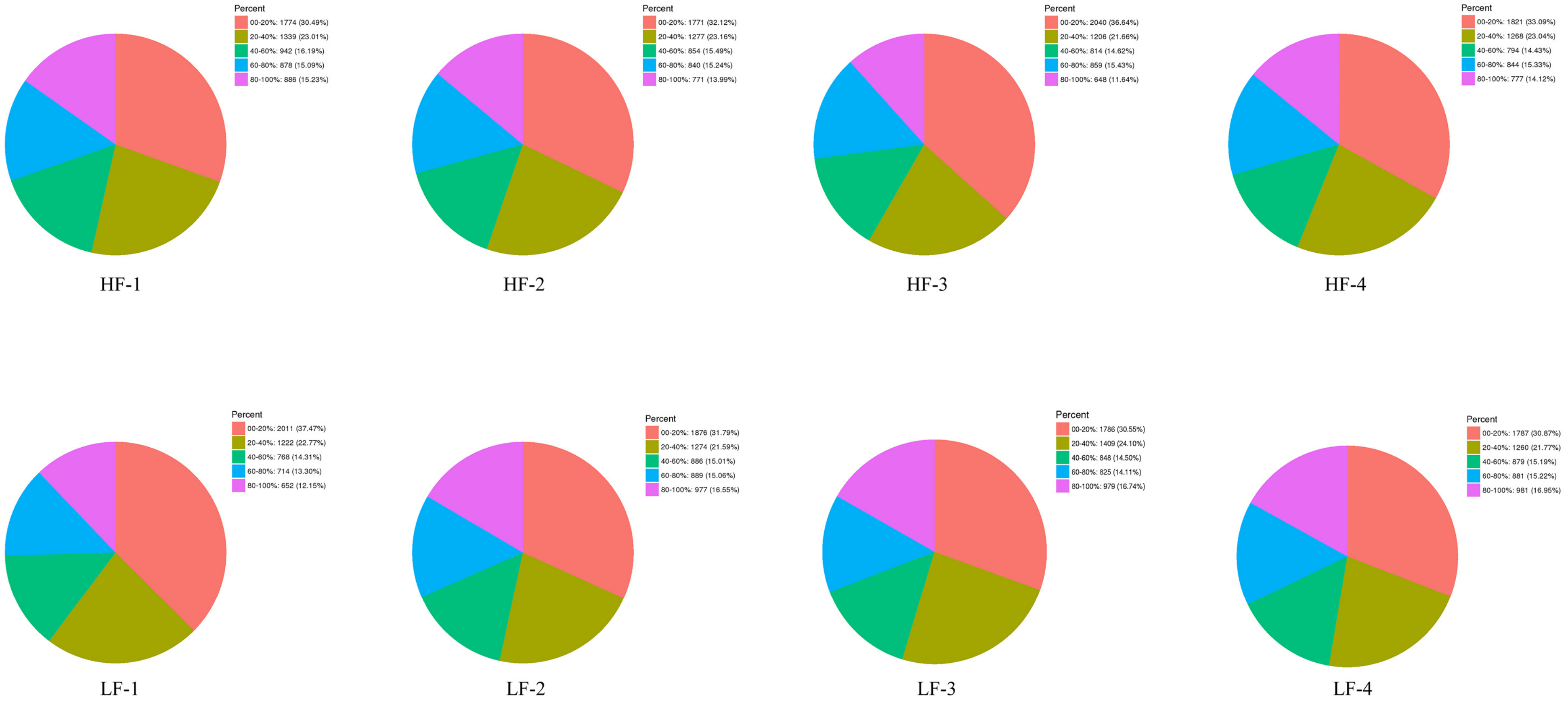

Figure S1. lncRNA transcript coverage statistics.

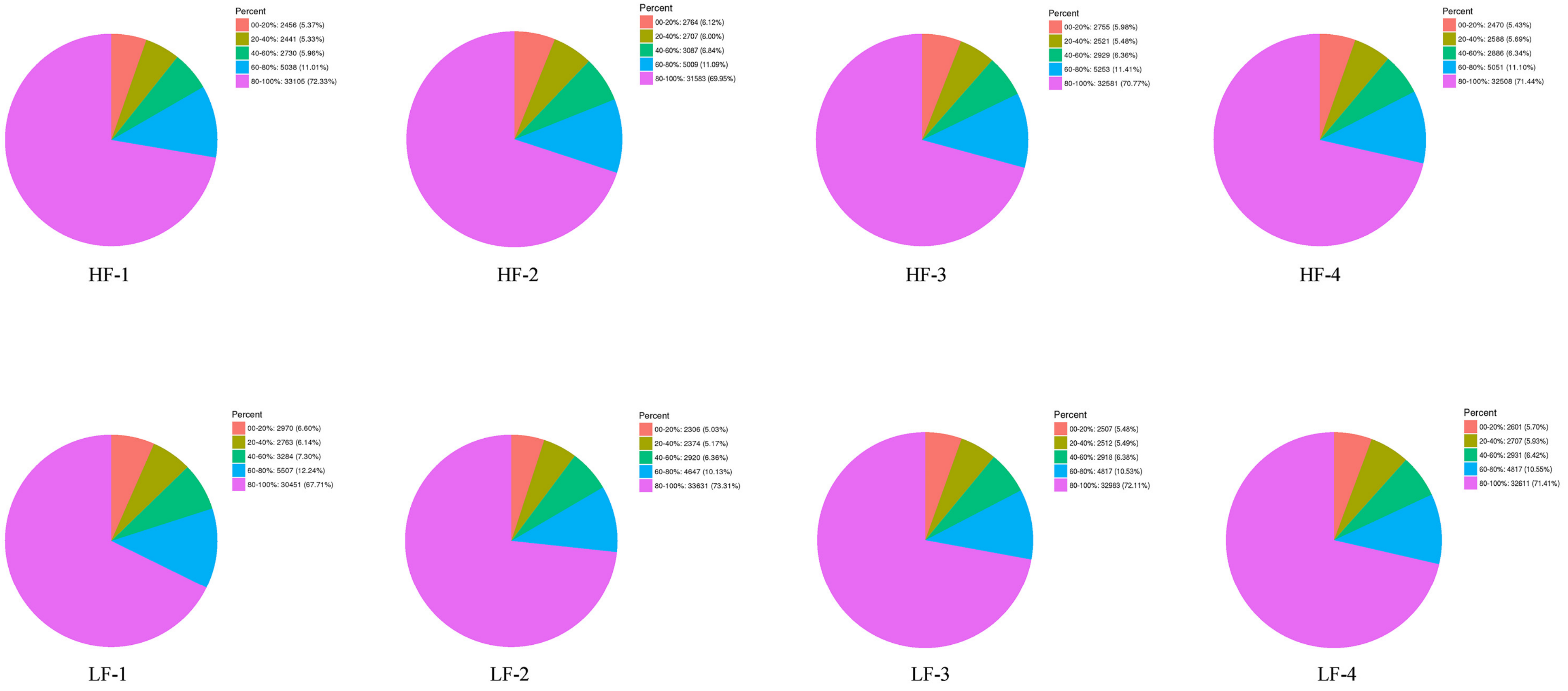

Figure S2. mRNA transcript coverage statistics.
